# Increased Arterial Stiffness in Prediabetic Subjects Recognized by Hemoglobin A1c with Postprandial Glucose but Not Fasting Glucose Levels

**DOI:** 10.3390/jcm8050603

**Published:** 2019-05-01

**Authors:** Chung-Hao Li, Feng-Hwa Lu, Yi-Ching Yang, Jin-Shang Wu, Chih-Jen Chang

**Affiliations:** 1 Department of Health Management Center, National Cheng Kung University Hospital, College of Medicine, National Cheng Kung University, Tainan 70403, Taiwan; smallhear@gmail.com; 2 Department of Family Medicine, National Cheng Kung University Hospital, College of Medicine, National Cheng Kung University, Tainan 70403, Taiwan; fhlu@mail.ncku.edu.tw (F.-H.L.); yichings@gmail.com (Y.-C.Y.); 3 Department of Family Medicine, College of Medicine, National Cheng Kung University, Tainan 70101, Taiwan

**Keywords:** prediabetes, arterial stiffness, baPWV

## Abstract

Previous studies exploring the association between arterial stiffness and prediabetes remain controversial. This study aimed to investigate the association of the different domains of prediabetes categorized by glycated hemoglobin A1c (A1c) 5.7–6.4%, impaired fasting glucose (IFG), fasting plasma glucose of 5.6–6.9 mmol/L, and impaired glucose tolerance (IGT), two-hour post-load glucose of 7.8–11.0 mmol/L, on arterial stiffness. These were measured by brachial–ankle pulse-wave velocity (baPWV). We enrolled 4938 eligible subjects and divided them into the following nine groups: (1) normoglycemic; (2) isolated A1c 5.7–6.4%; (3) isolated IFG; (4) IFG with A1c 5.7–6.4%; (5) isolated IGT; (6) combined IGT and IFG with A1c <5.7%; (7) IGT with A1c 5.7–6.4%; (8) combined IGT and IFG with A1c 5.7–6.4%; and (9) newly diagnosed diabetes (NDD). The baPWV values were significantly high in subjects with NDD (β = 47.69, 95% confidence interval (CI) = 29.02–66.37, *p* < 0.001), those with IGT with A1c 5.7–6.4% (β = 36.02, 95% CI = 19.08–52.95, *p* < 0.001), and those with combined IGT and IFG with A1c 5.7–6.4% (β = 27.72, 95% CI = 0.68–54.76, *p* = 0.044), but not in the other subgroups. These findings suggest that increased arterial stiffness was found in prediabetes individuals having an A1c 5.7–6.4% with IGT, but not IFG. Isolated A1c 5.7–6.4% and isolated IGT were not associated with elevated arterial stiffness.

## 1. Introduction

Type 2 diabetes mellitus is one of the leading causes of premature morbidity and mortality worldwide, and is an increasing public health concern [1]. Subjects with a condition lying between normoglycemia and dysglycemia are considered to be prediabetic, and these clinical conditions have been associated with the development of microvascular and macrovascular complications [2]. Since 2009, the American Diabetes Association (ADA) has revised the criteria for the detection of prediabetes and diabetes to include glycated hemoglobin A1c (A1c) levels as diagnostically relevant. This is in addition to pre-existing diagnostics establishing impaired fasting glucose (IFG) and impaired glucose tolerance (IGT) [3,4]. Accordingly, these ADA recommendations are based mainly on associations between A1c levels and microvascular complications [3].

Diabetes both precedes and contributes to the development of macrovascular disease [1], and the related diagnosis of prediabetes has become an important public issue [5]. One underlying mechanism of diabetes and macrovascular disease is the associated elevated serum advanced glycation end products (AGEs) that may cause the crosslinking of collagen molecules and the loss of collagen elasticity. The glycosylation of the vessel walls has also been related to the thinning of elastin fibers, subsequently resulting in arterial stiffness [6,7,8], which is an independent predictor of future cardiovascular events and mortality [9,10]. Several non-invasive methods are currently available to evaluate the severity of arterial stiffness, including one widely used method that measures pulse-wave velocity (PWV). Although carotid-femoral PWV (cfPWV) is considered the gold standard indicator of arterial stiffness, brachial–ankle PWV (baPWV) has been validated and is used in most Asian countries due to ease of execution, reproducibility, and the strong correlations with cfPWV [11,12].

Previous studies exploring the association between arterial stiffness and prediabetes have lacked consistency [13,14,15,16,17]; one explanation for the discrepancies might be related to the different ways of defining prediabetes, including determining the fasting plasma glucose (FPG), two-hour post-load glucose (2hPG), and A1c levels. For example, Ohnishi et al. [13] found significant differences in the baPWV values of the normal versus the IFG groups based on FPG level alone. In contrast, using both FPG and 2hPG level measurements, our previous study demonstrated the association of IGT with greater arterial stiffness and isolated IFG with insignificant differences in baPWV, compared with normal glucose tolerance [14]. Because 2hPG levels were not determined, the characteristics of the subjects in the IFG group of the study by Ohnishi et al. may have had similar characteristics to the subjects in the IGT group. Hence, the effects of IGT may have been misunderstood to be that of IFG. In addition, in a large study of Caucasian adults [18], the concordance of prediabetes, diagnosed via IFG, IGT, or A1c 5.7–6.4%, was limited, and, thus, the agreement among the three diagnostic criteria was only 10.4%. The relationship between arterial stiffness and prediabetes requires consensus among these three blood glucose indices. The aim of this study was to investigate the different domains of prediabetes categorized by A1c 5.7–6.4%, IFG, and IGT, as per the ADA criteria parameters of arterial stiffness shown by the baPWV values of a large Chinese population without known hypertension, diabetes, or cardiovascular disease.

## 2. Experimental Section

### 2.1. Study Population

Subjects who received self-motivated physical check-ups were included in the study, which covered the period between October 2006 and August 2009. The subjects’ data were extracted retrospectively for secondary data analysis, without personal identification information, from the Health Management Center of the National Cheng Kung University Hospital. None of the female participants were pregnant. To avoid confounding effects, individuals were excluded if they: (1) had a history of hypertension, diabetes, coronary heart disease, or stroke; (2) were using medications that are known to influence blood pressure, plasma glucose, and lipid profiles; (3) had either of their lower limbs amputated; (4) claimed alcohol consumption levels of more than 30 gm per week [19]; (5) had estimated glomerular filtration rates (eGFR) of <30 mL/min/1.73 m^2^; (6) were anemic (hemoglobin levels either <8.38 mmol/L in men or <7.45 mmol/L in women) [20]; or (7) had brachial–ankle indexes of <0.95 [21]. A total of 4938 subjects (3076 men, 62.3%; 1862 women, 37.7%) met the inclusion criteria and were enrolled. The study protocol was approved by the Ethics Committee for Human Research at the National Cheng Kung University Hospital, Taiwan (Approval No. ER-100-164).

### 2.2. Clinical Parameter Assessment

All participants completed a structured questionnaire containing questions regarding medical history, medication use, and lifestyle habits including smoking, alcohol consumption, and regular exercise. Smoking habits were categorized as former, current, or never; alcohol consumption was categorized as either current or non-user. Habitual exercise was defined as vigorous exercise at least three times per week [22]. The subjects’ height and weight were measured to the nearest 0.1 cm and 0.1 kg, respectively using a certified anthropometry instrument, and body mass indexes (BMI; kilograms per meter squared) were calculated for all subjects. Systolic and diastolic blood pressure (SBP and DBP, respectively) were determined using a DINAMAP vital sign monitor (model 1846SX, Critikon Inc., Tampa, FL, USA). The mean levels, from right and left, of brachial blood pressure were measured while the subjects were in the supine position and wrapped in appropriately-sized pneumatic pressure cuffs, after at least 15 min of rest.

**Figure 1:**
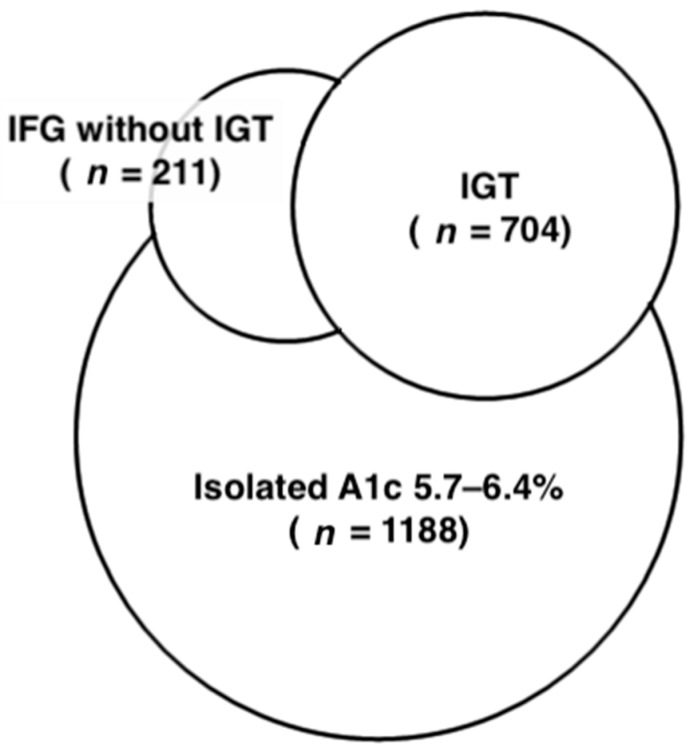
Venn diagram representing different domains of prediabetes categorized by glycated hemoglobin A1c (A1c) of 5.7–6.4%, impaired fasting glucose (IFG), and impaired glucose tolerance (IGT).

**Figure 2:**
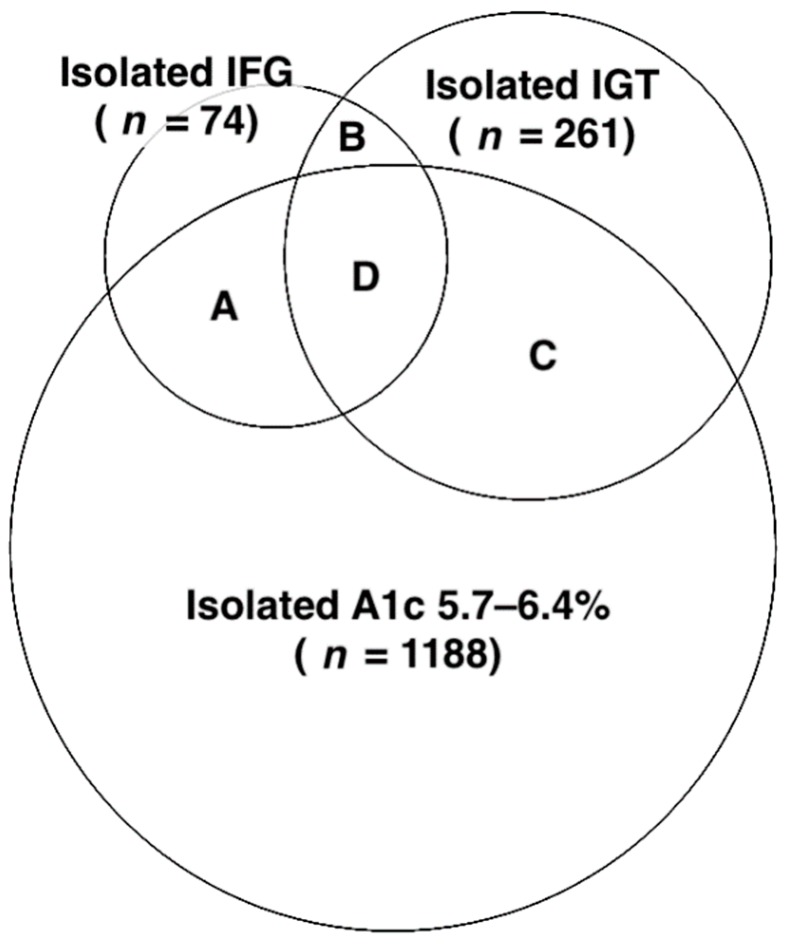
Venn diagram representing different domains and agreements of prediabetes categorized by glycated hemoglobin A1c (A1c) 5.7–6.4%, impaired fasting glucose (IFG), and impaired glucose tolerance (IGT). A) IFG with A1c 5.7–6.4% (*n* = 137); B) combined IGT and IFG with A1c <5.7% (*n* = 29); C) IGT with A1c 5.7–6.4% (*n* = 305); and D) combined IGT and IFG with A1c 5.7–6.4% (*n* = 109).

All blood samples were collected after overnight fasting of at least 10 h, and creatinine, FPG, cholesterol, triglyceride, high-density lipoprotein cholesterol (HDL-C), A1c, and hemoglobin levels were determined. Following a 75 g oral glucose tolerance test, 2hPG levels were measured in subjects without a history of diabetes. FPG and 2hPG levels were determined using a hexokinase method (Roche Diagnostic GmbH, Mannheim, Germany). A1c levels were measured using ion-exchange HPLC (HbA1c, BIO-RAD V-II TURBO Hemoglobin A1c program, Bio-Rad Laboratories, Inc., Kent, England). Initially, subjects were divided into five groups, according to the ADA diagnostic criteria [4]: (1) normoglycemic—FPG <5.6 mmol/L, 2hPG <7.8 mmol/L, and A1c <5.7%; (2) isolated A1c 5.7–6.4%—FPG <5.6 mmol/L, 2hPG <7.8 mmol/L, and A1c 5.7–6.4%; (3) IFG without IGT—FPG <7.0 mmol/L, 2hPG <7.8 mmol/L, and A1c <6.5%; (4) IGT—2hPG of 7.8–11.0 mmol/L, FPG <7.0 mmol/L, and A1c <6.5%; and (5) with newly diagnosed diabetes (NDD)—FPG ≥7.0 mmol/L, 2hPG ≥11.1 mmol/L, or A1c ≥6.5% (Figure 1). Groups 3 and 4 were further categorized into two and four groups, respectively: (3.1) isolated IFG—FPG of 5.6–6.9 mmol/L, 2hPG <7.8 mmol/L, and A1c <5.7%; (3.2) IFG with A1c 5.7–6.4%—FPG of 5.6–6.9 mmol/L, 2hPG <7.8 mmol/L, and A1c 5.7–6.4%; (4.1) isolated IGT—FPG <5.6 mmol/L, 2hPG of 7.8–11.0 mmol/L, and A1c <5.7%; (4.2) combined IGT and IFG with A1c <5.7%—FPG of 5.6–6.9 mmol/L, 2hPG of 7.8–11.0 mmol/L, and A1c <5.7%; (4.3) IGT with A1c 5.7–6.4%—FPG <5.6 mmol/L, 2hPG of 7.8–11.0 mmol/L, and A1c 5.7–6.4%; and (4.4) combined IGT and IFG with A1c 5.7–6.4%—FPG of 5.6–6.9 mmol/L, 2hPG of 7.8–11.0 mmol/L, and A1c 5.7–6.4% (Figure 2).

### 2.3. Vascular Assessment

After 5 min of supine rest, the subjects’ baPWV values were measured using a non-invasive vascular screening device (BP-203RPE II; Colin Medical Technology, Komaki, Japan). This device allows pulse-wave measurements from sensors in four wrapped pneumatic pressure cuffs that simultaneously measure the blood pressure and pulse waves in the brachial arteries of both arms and the tibial arteries of both legs. Then, we automatically computed baPWV (cm/s) by dividing the brachial–ankle distances (L = 0.5934 × body height (cm) + 14.4014) by the time interval between rising waveforms of the brachial region and the ankle (ΔT). Because the left and right baPWV values were significantly positively correlated (*r* = 0.968, *p* < 0.001), the mean baPWV values were used in the final analyses.

### 2.4. Statistical Methods

Statistical analyses were performed using SPSS software for Windows (version 17.0; SPSS, Inc., Chicago, IL, USA). Continuous variables are expressed as mean ± standard deviation, and categorical variables are expressed as percentages. Differences between the unadjusted group means of continuous variables were identified using ANOVA with Scheffé’s post hoc test. Categorical variables were compared using Chi-square tests among groups. Multiple linear regression analyses were performed to test the relationship between baPWV values (dependent variables) and different glycemic states, with adjustment for other confounding variables, including age, sex, BMI, SBP, total cholesterol, triglyceride, HDL, former smoking vs. never, current smoking vs. never, current alcohol consumption, and habitual exercise. In Models 1 and 2 of the multiple linear regression models, glycemic states were divided into five and nine subgroups, respectively. Unstandardized regression coefficients and 95% confidence intervals (CI) were derived from each regression model. Through the statistical tests, the differences and associations were considered significant when *p* < 0.05.

## 3. Results

Initially, 4938 subjects were classified into groups, normoglycemic (*n* = 2583), isolated A1c 5.7–6.4% (*n* = 1188), IFG without IGT (*n* = 211), IGT (*n* = 704), and NDD (*n* = 252). The Venn diagram representing different domains of prediabetes, including isolated A1c 5.7–6.4%, IFG without IGT, and IGT, is shown in Figure 1. The baseline characteristics of the subjects in these five groups are presented in Table 1. Significant differences were identified in age, sex, BMI, SBP, DBP, FPG, 2hPG, cholesterol, triglyceride, HDL-C, and prevalence of current alcohol drinking. Within these five groups, the mean baPWV values were 1253.1 ± 187.6, 1353.7 ± 223.9, 1376.9 ± 227.8, 1406.3 ± 251.7, and 1491.6 ± 276.6 cm/s (ANOVA; *p* < 0.001), respectively. The Scheffé’s post hoc test also revealed that baPWV values increased more in the isolated A1c 5.7–6.4%, IFG without IGT, IGT and NDD groups than in the normoglycemic group (*p* < 0.001). Following the discordance in the categorization resulting from the three tests (A1c, FPG, 2hPG), the prediabetic groups were further clarified, as shown in Figure 2, and all subjects were then classified into nine groups, including normoglycemic (*n* = 2583), isolated A1c 5.7–6.4% (*n* = 1188), isolated IFG (*n* = 74), IFG with A1c 5.7–6.4% (*n* = 137), isolated IGT (*n* = 261), combined IGT and IFG with A1c <5.7% (*n* = 29), IGT with A1c 5.7–6.4% (*n* = 305), combined IGT and IFG with A1c 5.7–6.4% (*n* = 109), and NDD (*n* = 252).

In the multiple linear regression analyses of the clinical variables and the baPWV values of Table 2, Model 1 shows independently higher baPWV values only in the prediabetic group of IGT (β = 16.59, 95% CI = 4.41–28.76, *p* = 0.008) and the NDD group (β = 46.35, 95% CI = 27.18–65.04, *p* < 0.001), but not in the isolated A1c 5.7–6.4% or IFG without IGT groups, compared with normoglycemic subjects. In addition, age, current smoking, and SBP were positively associated with the baPWV values, whereas BMI and habitual exercise were inversely associated with the baPWV values. These above factors accounted for 63.2% of the total variance (adjusted *R*^2^ = 0.632). In Model 2, when the agreement domains were stratified further using the three tests, the baPWV remained significantly higher in the NDD group (β = 47.69, 95% CI = 29.02–66.37, *p* < 0.001) and the prediabetic groups of IGT with A1c 5.7–6.4% (β = 36.02, 95% CI = 19.08–52.95, *p* < 0.001) and combined IGT and IFG with A1c 5.7–6.4% (β = 27.72, 95% CI = 0.68–54.76, *p* = 0.044). However, compared to the normoglycemic subgroup, the elevated baPWV values were not significant in the two subgroups—isolated and combined IGT and IFG with A1c <5.7%—originally included from the IGT group. The above factors accounted for 63.3% of the total variance (adjusted *R*^2^ = 0.633).

## 4. Discussion

The present study is the first to show the different effects of prediabetes, categorized concomitantly by A1c, FPG, and 2hPG levels, on arterial stiffness. The results showed that increased arterial stiffness was found in individuals having either IGT with A1c 5.7–6.4% or combined IGT and IFG with A1c 5.7–6.4% but not in those having either IGT with A1c <5.7% or combined IGT and IFG with A1c <5.7%, independent of other cardiovascular risk factors. The elevated arterial stiffness was insignificant in subjects having either IFG with A1c <5.7% or IFG with A1c 5.7–6.4%. The association between prediabetes and arterial stiffness is still not consistent [13,14,15,16,17]. The MARK study [17] showed that A1c, FPG, and postprandial glucose were positively related to arterial stiffness in diabetes subjects, but not in prediabetes subjects. Ohnishi et al. [13] found that baPWV values were significantly higher than normal fasting glucose levels in IFG subjects; however, they only had fasting glucose data and not 2hPG data. Our previous study highlighted the importance of IGT, categorized by FPG and 2hPG values, and the associated risk of increased arterial stiffness based on FPG and 2hPG levels [14]. Di Pino et al.’s study [16] revealed that arterial stiffness, as shown by an augmentation index, was higher in subjects with normal glucose tolerance with A1c 5.7–6.4% compared to that in subjects with normal glucose tolerance with A1c <5.7%; however, these values were similar to those of the IGT and type 2 diabetes patients. Liang et al. [15] found that prediabetic subjects with abnormal A1c, FPG, and 2hPG had higher cfPWV values, but they did not test the concomitant influence of A1c, FPG, and 2hPG on cfPWV. The discrepancy between the results of this study and those of Liang et al. may be related to the different methodologies. Subjects were excluded from our study if they had history of hypertension, diabetes, coronary heart disease, stroke, or peripheral vascular disease and if they were taking medications known to influence blood pressure, plasma glucose, and lipid profiles, whereas such subjects were not excluded from Liang et al.’s study. In Liang et al.’s study, subjects with cardiovascular disease or those taking medications were included among subjects with A1c 5.7–6.4%, IFG, or IGT, resulting in elevated arterial stiffness in subjects with abnormal A1c, FPG, and 2hPG.

Discrepancies in the criteria used in the three different tests for diagnosing either prediabetes or diabetes have been reported and suggest the importance of categorizing the different characteristics of the subjects. IFG indicates impaired first-phase insulin secretion and reduced hepatic insulin sensitivity, whereas IGT suggests markedly peripheral insulin resistance and defective second-phase insulin secretion, contributing to prolonged defects [23]. A1c is a marker representing both basal and postprandial hyperglycemia, and it provides a “picture” of the average blood glucose level over the past 2–3 months [24]. In this study, increased arterial stiffness was found in prediabetes individuals having an A1c 5.7–6.4% with IGT, but not IFG. This result highlighted the importance of 2hPG, as possibly having a stronger associated risk of arterial stiffness than that of FPG. The main mechanism of hyperglycemia affecting arterial stiffness is the generation and formation of AGEs, whose biochemical process may involve the glycosylation of vessel walls, subsequent crosslinking of collagen molecules, loss of collagen elasticity, and thinning of elastin fibers. Otherwise, AGEs can interact with certain receptors, inducing intracellular signaling with enhanced oxidative stress, triggering the inflammatory process, and, finally, increasing arterial stiffness [6,7,8,25,26]. Furthermore, the serum levels of AGEs are positive correlates of insulin resistance [27], which is related to the reduced production of nitric oxide and its related vasodilation [28], post-load hyperinsulinemia-induced oxidative stress [29], decreased endothelial progenitor cells with impaired vascular repair qualities [30], and related metabolic alterations, such as either dyslipidemia or elevated blood pressure [31]. Importantly, IGT showed a more pronounced degree of insulin resistance than did IFG [32]. Therefore, the above findings may partially explain elevated arterial stiffness in prediabetes individuals having an A1c 5.7–6.4% with IGT, but not IFG.

As shown previously, we have identified the independent effects of age, blood pressure, and smoking status on arterial stiffness [33,34,35]. Aging is widely associated with compromised arterial stiffness; the effects of aging on baPWV might be mediated by the intermediate parameters of cardiovascular risk factors, such as blood pressure [36]. The positive relationship between smoking and increased arterial stiffness may reflect increased inflammation, thrombosis, oxidation of low-density lipoprotein cholesterol, and oxidative stress [34]. Although the association between obesity and arterial stiffness remained inconclusive, in agreement with Tomiyama et al. [37], BMI was negatively associated with baPWV in this study. In the work of Ben et al. [38], arterial stiffness was reduced until middle age, and it was speculated that vascular adaptation to obesity was lost with advanced age, meaning that the adverse association between obesity and arterial stiffness becomes apparent only later in life. Moreover, the effects of obesity on arterial stiffness might be mediated by intermediate cardiovascular risk factors, such as blood pressure, and the highly associated variables included might lead to further collinearity [39].

Despite the large study cohort, our study was limited to a cross-sectional design, which cannot be used to establish causal relationships. Our study was also confined to the Chinese population, and the data complemented the differences between previous studies of different ethnic groups. All subjects were extracted from a health management center and had received regular health examinations. Therefore, the present results should be used carefully because they are not indicative of the general population. In addition, since the arterial stiffness in our study was measured by baPWV, a peripheral stiffness parameter but not a direct measure of central stiffness, our results are limited and would be more relevant if the subjects were then used for a longitudinal study, either in terms of future cardiovascular events or mortality. Additionally, we did not measure either insulin or high sensitivity C-reactive protein to provide more information about insulin resistance or vascular inflammation status.

## 5. Conclusions

In conclusion, increased arterial stiffness was found in prediabetes individuals having an A1c 5.7–6.4% with IGT, but not IFG. Isolated A1c 5.7–6.4%, isolated IGT, and IGT with A1c <5.7% were not associated with elevated arterial stiffness. Despite the practicality and convenience of FPG and A1c, it is still important to consider postprandial glucose values to facilitate early recognition of the significant proportion of the at-risk population.

## Figures and Tables

**Table 1:** Demographic and clinical characteristics of study subjects with differing glycemic statuses according to A1c, FPG, and 2hPG.

	Normoglycemic ^1^	Prediabetes			NDD ^5^	*p* *	Post Hoc Test **
		Isolated A1c 5.7–6.4% ^2^	IFG without IGT ^3^	IGT ^4^			
Variables	(*n* = 2583)	(*n* = 1188)	(*n* = 211)	(*n* = 704)	(*n* = 252)		
Age (years)	42.3 ± 10.7	49.6 ± 10.5	50.0 ± 11.0	50.8 ± 11.2	53.6 ± 11.1	<0.001	a, b, c, d, g, i, j
Gender, male (%)	59.5	62.4	65.9	69.7	66.3	<0.001	-
Body mass index (kg/m^2^)	23.3 ± 3.3	24.4 ± 3.3	25.5 ± 2.9	25.0 ± 3.5	25.7 ± 3.6	<0.001	a, b, c, d, e, f, g
Former/Current smoking (%)	5.2/9.6	5.8/9.8	7.1/10.9	8.0/9.8	9.1/9.1	0.115	-
Current alcohol drinking (%)	10.5	9.6	12.3	14.1	12.3	0.029	-
Habitual exercise (%)	7.0	8.0	6.2	6.1	4.4	0.230	-
SBP (mmHg)	113.5 ± 12.8	118.1 ± 13.6	122.4 ± 13.8	121.9 ± 15.2	125.8 ± 14.8	<0.001	a, b, c, d, e, f, g, j
DBP (mmHg)	67.4 ± 9.8	70.9 ± 9.8	73.8 ± 9.9	73.4 ± 10.3	76.1 ± 10.1	<0.001	a, b, c, d, e, f, g, j
FPG (mmol/L)	4.67 ± 0.37	4.84 ± 0.37	5.77 ± 0.22	4.96 ± 0.63	6.92 ± 2.99	<0.001	a, b, c, d, e, f, g, h, i, j
2hPG (mmol/L)	5.29 ± 1.13	5.64 ± 1.16	5.98 ± 1.13	8.93 ± 0.87	13.78 ± 4.97	<0.001	a, b, c, d, e, f, g, h, i, j
Cholesterol (mmol/L)	4.96 ± 0.89	5.27 ± 0.96	5.34 ± 0.92	5.28 ± 0.90	5.58 ± 1.06	<0.001	a, b, c, d, g, j
Triglyceride (mmol/L)	1.24 ± 0.74	1.43 ± 0.86	1.75 ± 1.22	1.69 ± 1.11	1.90 ± 1.19	<0.001	a, b, c, d, e, f, g, j
HDL-C (mmol/L)	1.39 ± 0.38	1.31 ± 0.36	1.28 ± 0.34	1.23 ± 0.33	1.22 ± 0.34	<0.001	a, b, c, d, f, g
baPWV (cm/sec)	1253.1 ± 187.6	1353.7 ± 223.9	1376.9 ± 227.8	1406.3 ± 251.7	1491.6 ± 276.6	<0.001	a, b, c, d, f, g, i, j

Data presented as means ± standard deviation or percentage (%); ^1^ Group 1: normoglycemic (A1c <5.7%, FPG<100 mg/dL, and 2hPG <140 mg/dL); ^2^ Group 2: isolated A1c 5.7–6.4% (A1c 5.7–6.4%, FPG<100 mg/dL, and 2hPG < 140 mg/dL); ^3^ Group 3: IFG without IGT (FPG 100–125 mg/dL, 2hPG <140 mg/dL, and A1c <6.5%); ^4^ Group 4: IGT (FPG <126 mg/dL, 2hPG 140–199 mg/dL, and A1c <6.5%); ^5^ Group 5: NDD (FPG ≥126 mg/dL, or 2hPG ≥200 mg/dL, or A1c ≥6.5%); IFG and IGT was not included in isolated A1c 5.7–6.4%; * *p* for the difference, between the five groups, by ANOVA; ** Scheffé’s tests: a—1 vs. 2; b—1 vs. 3; c—1 vs. 4; d—1 vs. 5; e—2 vs. 3; f—2 vs. 4; g—2 vs. 5; h—3 vs. 4; i—3 vs. 5; j—4 vs. 5. Abbreviations: A1c—glycated hemoglobin A1c; IFG—impaired fasting glucose; IGT—impaired glucose tolerance; NDD—newly diagnosed diabetes; SBP—systolic blood pressure; DBP—diastolic blood pressure; FPG—fasting plasma glucose; 2hPG—two-hour post-load glucose; HDL-C—high-density lipoprotein cholesterol; baPWV—brachial–ankle pulse-wave velocity.

**Table 2:** Coefficients (β) and 95% confidence intervals (CI) for the independent effects of clinical variables on baPWV level.

Independent Variables	Model 1		Model 2	
	β (95% CI)	*p* Value	β (95% CI)	*p* Value
Age (years)	−	<0.001	8.27 (7.89~8.65)	<0.001
Gender (female vs. male)	5.39 (−4.00~14.77)	0.260	5.02 (−4.36~14.39)	0.294
Body mass index (kg/m^2^)	−8.09 (−9.41~−6.77)	<0.001	−8.20 (−9.52~−6.87)	<0.001
Smoking (former vs. never)	8.16 (−8.77~25.08)	0.345	8.60 (−8.32~25.52)	0.319
Smoking (current vs. never)	20.56 (6.42~34.71)	0.004	19.73 (5.59~33.87)	0.006
Current alcohol drinking (yes vs. no)	-8.51 (−22.03~5.01)	0.217	−8.13 (−21.65~5.39)	0.238
Habitual exercise (≥3 times/week vs. <3 times/week)	−22.12 (−37.26~−6.98)	0.004	−22.47 (−37.61~−7.34)	0.004
Cholesterol (mmol/L)	1.81 (−2.84~6.45)	0.445	1.59 (−3.04~6.24)	0.500
Triglyceride (mmol/L)	3.98 (−1.23~9.19)	0.134	3.81 (−1.41~9.02)	0.152
HDL-C (mmol/L)	−9.96 (−23.79~3.87)	0.158	−9.47 (−23.92~4.36)	0.180
SBP (mmHg)	9.06 (8.74~9.37)	<0.001	9.04 (8.72~9.36)	<0.001
Isolated A1c 5.7–6.4% * vs. normoglycemic	4.84 (−5.08~14.75)	0.339	5.56 (−4.36~15.47)	0.272
IFG without IGT vs. normoglycemic	−7.52 (−27.21~12.17)	0.454	−	−
Isolated IFG vs. normoglycemic	−	−	−14.36 (−46.13~17.40)	0.375
IFG with A1c 5.7–6.4% vs. normoglycemic	−	−	−2.32 (−26.41~21.78)	0.851
IGT vs. normoglycemic	16.59 (4.41~28.76)	0.008	−	−
Isolated IGT vs. normoglycemic	−	−	−6.90 (−24.55~10.74)	0.443
Combined IGT and IFG with A1c <5.7% vs. normoglycemic	−	−	4.51 (−45.88~54.91)	0.861
IGT with A1c 5.7–6.4% vs. normoglycemic	−	−	36.02 (19.08~52.95)	<0.001
Combined IGT and IFG with A1c 5.7–6.4% vs. normoglycemic	−	−	27.72 (0.68~54.76)	0.044
NDD vs. normoglycemic	46.35 (27.18~65.04)	<0.001	47.69 (29.02~66.37)	<0.001
Adjusted *R*^2^ (%)	63.2		63.3	

* IFG and IGT were not included in isolated A1C 5.7–6.4%. Abbreviations: baPWV—brachial–ankle pulse-wave velocity; HDL-C—high-density lipoprotein cholesterol; SBP—systolic blood pressure; A1c—glycated hemoglobin A1c; IFG—impaired fasting glucose; IGT—impaired glucose tolerance; NDD—newly diagnosed diabetes.
